# Assessing Movements of Brushtail Possums (*Trichosurus vulpecula*) in Relation to Depopulated Buffer Zones for the Management of Wildlife Tuberculosis in New Zealand

**DOI:** 10.1371/journal.pone.0145636

**Published:** 2015-12-21

**Authors:** Andrea E. Byrom, Dean P. Anderson, Morgan Coleman, Caroline Thomson, Martin L. Cross, Roger P. Pech

**Affiliations:** Landcare Research, P.O. Box 69040, Lincoln 7640, New Zealand; University of Tasmania, AUSTRALIA

## Abstract

In New Zealand, managing the threat of bovine tuberculosis (TB) to livestock includes population reduction of potentially infectious wildlife, primarily the brushtail possum (*Trichosurus vulpecula*). Population control is often targeted on forested buffer zones adjacent to farmland, in order to limit movements of possums across the buffer and reduce the risk of disease transmission to livestock. To assess the effectiveness of buffers in protecting livestock we analysed GPS telemetry data from possums located in untreated forest adjacent to buffers, and used these data to characterise patterns of movement that could lead to possums reaching farmland during the season when most dispersal occurs. Analyses of movement data showed that the direction of dispersal by sub-adult and adult possums and the extent of long exploratory movements were not biased toward forest buffers, even though these provided vacant habitat as suitable for possums as untreated forest. Instead, dispersal and exploratory movements were uncommon even for sub-adult possums and such events typically lasted <10 days. Dispersing possums settled predominantly in river valleys. A simulation model was developed for the 3-6-month dispersal season; it demonstrated a probability of <0.001 that an infected possum, originating from a low-density population with low disease prevalence in untreated forest, would move across 3 km of recently controlled forest buffer to reach farmland. Our results indicate short-term reduction in the risk of TB transmission from possums to livestock in New Zealand by the use of depopulated buffer zones, while acknowledging that the threat of disease spread from untreated forest is likely to increase over time as possum population density and, potentially, TB prevalence among those possums, increase in the buffer zone.

## Introduction

The agricultural industry in New Zealand, in common with that in the British Isles, has experienced outbreaks of bovine tuberculosis (TB) attributable, at least in part, to transmission of *Mycobacterium bovis* infection from a ubiquitous wildlife reservoir host [1,2]. In New Zealand this is due to an introduced marsupial, the brushtail possum (*Trichosurus vulpecula*). Unlike badgers (*Meles meles*), the principal wildlife TB host in the British Isles, possums in New Zealand are not subject to species protection legislation, and accordingly this recurrent source of infection has been controlled with the aid of population reduction [3]. This approach, in combination with conventional farming practices for TB management, has seen New Zealand’s annual livestock herd reactor rate decline by >95% over the last 20 years [4].

Brushtail possums are predominantly arboreal, 2–4 kg generalist browsers and opportunistic omnivores; in New Zealand they can reach population densities exceeding 10 animals/ha in the most preferred forested habitats where individual animals’ home ranges are usually <5ha [5,6]. As a TB vector, and also a conservation threat, possums have been subject to control measures since the 1970s. Mostly, this has been achieved through trapping and poisoning to reduce the local possum population to a point where it is no longer a reservoir for *M*. *bovis* [7]. However, in practicality, there are many instances where possum control cannot be maintained for long enough, or at sufficient intensity, to eliminate intra-specific *M*. *bovis* transmission. This has led to an alternative strategy that utilises buffer zones to isolate infected wildlife populations from TB-susceptible livestock [8]. A buffer zone may be created either by removing potential wildlife hosts (i.e. lethal control [9]) or by vaccination of wildlife to reduce the pool of susceptible hosts [10,11]. Depopulated buffer zones have been used in New Zealand for many years [12] while vaccine-protected buffer zones for wildlife remain theoretical [13].

For the foreseeable future, TB management in some places in New Zealand will rely on continued expenditure to maintain buffer zones. The cost per control operation is directly proportional to the area of those buffers. Accordingly, buffer widths should be as small as possible to minimise the costs of control. Another cost-reduction measure would be to expend greatest control effort in areas where long-distance movement by possums is most likely. In relation to buffer zones these are unknown; however Arthur et al. [14] suggested that the vacant habitat of possum-depopulated zones might attract possums into it by changes in the animals’ movement patterns in response to low-density environments. Possum movement from an untreated area (typically high possum density) into or through an adjacent buffer zone (zero to low density) is most likely to occur during the annual (or biannual [9]) dispersal of sub-adult animals [5]. In addition, landscape attributes (e.g. river courses, gorges or high ridges) might restrict or channel possum movements and thus modify movement patterns. In this latter regard a recent study quantified the effort cost to possums of dispersing across a heterogeneous landscape [15], identifying rivers and elevation gradients as potential modifiers of movement.

In this study we used GPS telemetry data from possums in areas adjacent to forest buffer zones to assess movement patterns during the period when possums were most likely to disperse or make long exploratory movements [5]. Specifically, we addressed three questions. First, what proportion of possums exhibited movement patterns typified as: (1) settled home range; (2) home range displacement (i.e. a gradual shift in home range); (3) exploratory movements; or (4) dispersal to a new home range? Second, were the possums’ dispersal or exploratory movements influenced by an elevation gradient or by encountering a water-course? Third, how was the probability of incursion of an *M*. *bovis*-infected possum onto farmland influenced by the width of the buffer and by the population density and prevalence of TB among possums in adjacent un-controlled forest?

## Methods

### Ethics statement

Ethical approval to conduct this study was granted by Landcare Research Animal Ethics Committee (Lincoln, New Zealand; ethics approval number 09/09/02). Permission to conduct field work on public land was granted by New Zealand's Department of Conservation (DOC; www.doc.govt.nz).

### Study sites

The study was conducted in two areas of on-going possum control (Vector Control Areas, VCAs) in the West Coast region of New Zealand’s South Island. Three sites were selected in 2010 in the Upper Ahaura VCA (Haupiri River, Waikiti Hut, Waiheke River; 42.6S, 171.9E), and two sites were selected in 2011 in the Whataroa–Waitangi VCA (Whataroa River–Alf Creek, Wanganui River–Tribute Creek; 43.2S, 170.5E). All sites comprised mixed beech–kāmahi–podocarp forest [16] on variable inclines to an elevation of ~600m above glacial river valleys, with small clearings of introduced pasture grasses on the river flats (vegetation types described in detail in Byrom [17]). All sites included a major river flowing from forest to farmland; the rivers were wide (> 10 m) and likely presented a barrier to possum movement, although it is recognised that possums can, under exceptional circumstances, occasionally traverse water-courses [18].

Possum densities in the extensive mixed forests at the study sites are typically high (>6 possums/ha [6]) and TB is endemic among possums there [19,20]. Following standard practice for possum control, the main TB control agency (TBfree Ltd) had created possum-depleted buffer zones approximately 3km wide in the forested areas adjacent to open farmland at each of the study sites. Possum control had been conducted, in each case, during the winter immediately prior to data collection periods that began in mid-summer. Control comprised aerial deployment of 1080 toxin in pelleted cereal bait [21]. Additionally, ground-based control (trapping and poisoning) was undertaken annually along the immediate forest–farmland margins in both areas [8]. The efficacy of the combined aerial and ground operations was measured by post-control monitoring of possums using Trap-Catch Indices (TCIs) [8,22]: at two sites in the Upper Ahaura VCA in 2010 TCIs were <1.5% and <0.5% (TBfree vector monitoring operations, unpublished data; see supplementary information [S1 File]), indicating extremely low residual densities of possums of <0.5 animals/ha [23,24].

### Field methods

Beginning in late January and February of each year, just before the peak dispersal period for sub-adult animals [25], possums were caught at each of the five study sites using soft-catch leg-hold traps set in the untreated forest within 300 m of the control area boundaries. In the first year, 58 Sirtrack^TM^ GPS/VHF collars [26] were fitted to possums (20, 20 & 18 collars, respectively, at each of the three Upper Ahaura sites). In the second year 45 GPS/VHF collars were fitted to possums (24 & 21 collars, respectively, at each of the Whataroa–Waitangi sites). At each individual site, we aimed to place 35–50% of the available collars on sub-adult animals, as they are the age group thought most likely to disperse [27].

The GPS units were configured to acquire data overnight only (see below); possums are predominantly nocturnal and undertake most movement during darkness, remaining in one of several alternative den sites within their home range during daylight hours [5,28]. The movements of each possum were recorded for up to 4.5 months in each study year during the main dispersal period, with each GPS collar programmed to take three (first-year settings) or two (second-year settings) GPS fixes at 4-hourly intervals commencing at 9pm-11pm. In addition to automated data collection, manual GPS data were collected at approximately monthly intervals by first locating possums via conventional ground or aerial VHF telemetry. At completion of the study, collars were recovered from possums following poisoning or trapping and data were downloaded. Data were obtained from 82 of the 103 originally-deployed collars: 30 sub-adult possums (14 males, 16 females) and 52 adult possums (24 males, 28 females; and see supplementary information [S1 File]).

### Analysis of GPS data

For each animal we used a single location for each night that data were available. In cases where multiple locations were available for a night, we calculated the weighted average location using the inverse of the satellite-derived horizontal dilution of precision (HDOP: location accuracy index) as the weighting measure. Retaining all GPS data, including those with high HDOP values (i.e. slightly lower quality locational data [29]), was justified because this analysis used a single location to represent space use on a given night to assess movements over weeks and months, and at spatial scales similar to or greater than a possum’s home range. Also, our decision to use all GPS data for analyses of long-distance movements was based on a previous assessment of the relationship between HDOP and on-ground locational error in similar forest habitat in New Zealand [13]. We then plotted over time the squared displacement (see below) from the first location for each animal. We also assessed, via examination of plotted graphs and by cross-referencing to the GPS co-ordinates of the animals’ locations, how movements of possums varied in relation to rivers, topography, and between age and sex classes.

### Interpretation of possum movement patterns

In the first instance, if a possum’s squared-displacement distance did not increase over time, it was classified as exhibiting a settled home range. A second pattern of animal movement was when the squared displacement increased linearly (which has been reported to occur if an animal’s home range displacement occurs gradually via a diffusion or correlated random-walk process [30,31]). A third movement pattern, termed exploratory behaviour, was characterised by a pattern generally similar to a settled home range (i.e. no trend for changes over time in the squared-displacement distance) but interspersed with occasional forays over much longer distances. Finally, when the squared displacement increased in a highly non-linear way (i.e. typically a rapid increase followed by little or no further change), the possum was considered to be exhibiting dispersal and re-settlement [32–34].

### Predicted probability of incursion

**Fig 1:**
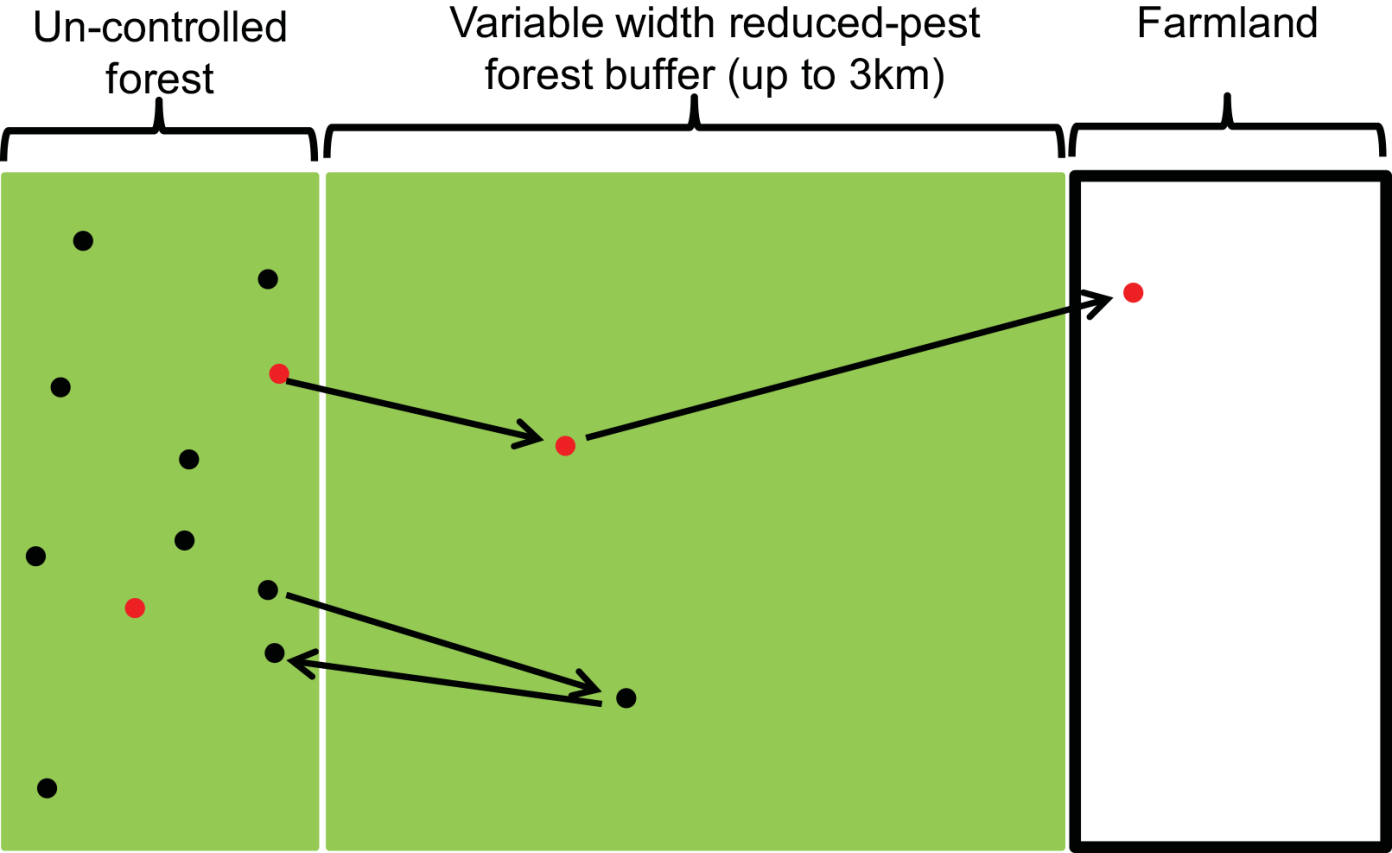
Schematic illustration of simulations used to determine the probability of an *M*. *bovis*-infected possum arriving in farmland by traversing a forested buffer. Initially, possums were placed randomly in an un-controlled forest block (black dots are locations of uninfected possums; red are infected). Arrows represent hypothetical examples of subsequent dispersal and exploratory movements.

We developed a simulation model written in the Python programming language [35] to explore how the probability of incursion onto farmland of at least one *M*. *bovis*-infected possum (a ‘TB incursion event’) would be influenced by the width of the buffer and by the population density and disease prevalence (i.e. percentage of infected possums) in adjacent un-controlled forest (Fig 1). We simulated 6 buffer widths, 2 possum population densities, and 2 disease-prevalence levels, which resulted in 24 simulation scenarios. The simulated un-controlled forested area with an infected possum population was a 3 km x 0.5 km forest block (infected block) with the long side adjacent to the recently controlled buffer. The number of infected possums in the infected block was the product of the density, area (1.5 km^2^), and TB prevalence. Population density values were set at typical high and low levels of 9 and 2 animals per ha for possums in this type of habitat [36]. The TB prevalence was set at levels of 0.10 or 0.02, typical of high and low values for endemic disease regions in this forest type [37]. The six buffer widths ranged between 500–3000 m in 500m increments. We repeated each simulation 1,000 times to obtain the probability of a TB incursion across the buffer (Fig 1), which was calculated as the number of iterations with at least one infected possum reaching farmland divided by the total number of iterations.

The starting locations of infected possums in a simulated population were placed randomly in the 1.5 km^2^ infected forest block. Long-distance movements, particularly exploratory movements and dispersal, are most likely to result in an infected possum reaching farmland. Based on the GPS data, these movements typically occur over periods of time of approximately 4–10 days, although within this period there can be a mix of short- and long-distance movements. Therefore, for each infected possum we simulated a single ‘representative movement event’, in which animals could exhibit the four characteristic behaviours evident from empirical data (settled home range, home range expansion, exploratory movement, or dispersal). We assessed the sensitivity of the estimated probability of an incursion event involving an infected possum to the number of days per representative movement event by repeating the simulation three times; representing a short 4-day, medium 7-day (most likely) and long 10-day event.

The probability of an infected individual reaching the farmland during the 3–6 month dispersal period was the joint probability of the individual travelling the required distance and direction. The movement trajectory was simulated as follows. For each simulated infected possum, we sampled from the entire movement dataset (step lengths and directions) a randomly selected individual. Assuming that our sample of collared possums represents the real population, random selection of an individual incorporates the probability of sampling dispersal or exploratory movements, the types of movement most likely to lead to a TB incursion onto farmland. From the movement data of the selected individual we then randomly selected, with replacement, step lengths and directions in pairs for the number of days in the representative movement event. Beginning at the starting location, we calculated the location of the simulated individual on each day and whether or not an incursion occurred. Our random sampling of the observed movement data made the assumption that a simulated animal was capable of all moves in any sequence. The lack of a modelled attraction to a home range centre made this simulation a random walk over the representative movement event and therefore produced an upper bound to the estimated probability of a TB incursion.

### Data accessibility

Data presented in this study are publicly accessible at http://datastore.landcareresearch.co.nz/dataset/raw-data-for-byrom-et-al-assessing-movement/resource/028c6177-ca1a-49e4-92b7-cfbb85d38559. The data sets comprise 1] raw GPS information downloaded from the possum GPS units, and 2] data used from possum movement patterns in model simulations to predict the likelihood (probability) of incursion of an *M*. *bovis*-infected possum onto adjacent farmland.

## Results

### Possum characteristics

Thirteen of the original 103 possums to which collars had been fitted could not be tracked, either because the collars slipped off the animal soon after fitting (n = 10) or due to malfunction in the VHF component of the collar (n = 3). Two of the remaining 90 possums died prior to completion of the tracking period, while six animals were known from tracking to be alive for most of the duration of the two study periods but were not re-captured or poisoned at completion. Thus possum survival was at least 92% and possibly as high as 98% over the 3–5 months from mid-summer in both years of the study. No overt TB was detected among the possums in this study, either at the time of collar fitting or at the time of carcass/collar recovery.

In total, 82 collars were recovered to access GPS data. Only possums with 8 or more days of movement data (i.e. the minimum for reliably characterising home range movements of possums [38]) were used for subsequent analysis. These comprised 79 possums (46 and 33 from the Upper Ahaura VCA in 2010 and the Whataroa–Waitangi VCA in 2011, respectively). Demographic details of these animals are provided in the supplementary information [S1 File].

### Patterns of movement by possums

From analysis of the GPS data, we found no evidence that the directions of dispersal and exploratory movements were biased towards forest with reduced possum density (i.e. preferential movement of possums into vacant territory), even though these provided habitat as suitable for possums as untreated forest. Further, we found no evidence that forested ridgelines changed movement patterns of possums, although our data did provide an indication that large rivers could act as barriers, resulting in possums moving primarily along valleys.

**Fig 2:**
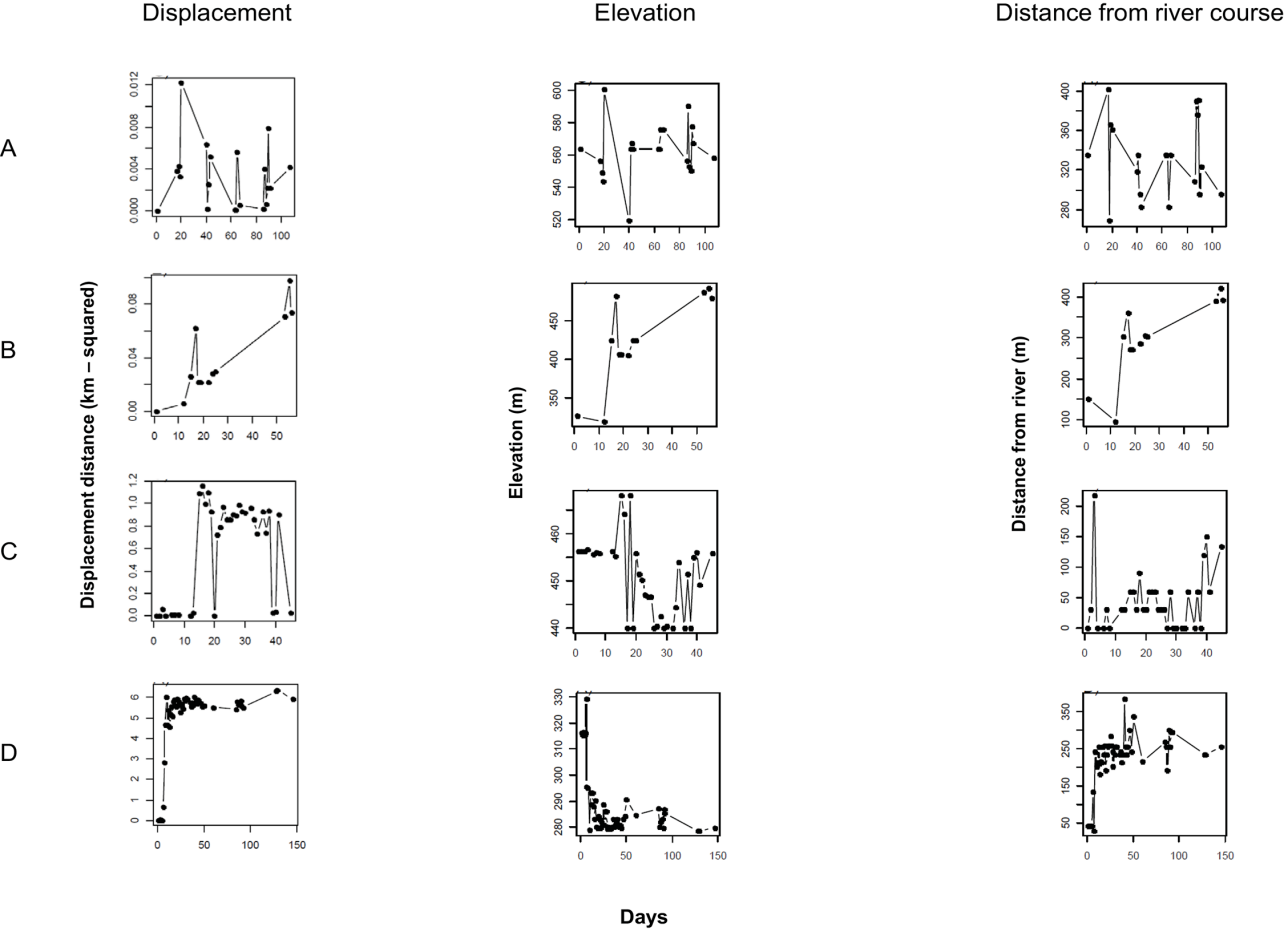
Example of changes in possum movement patterns over time. Data represent changes in the squared displacement over time (left hand column), and changes in elevation and distance from the main river at the Upper Ahaura site (right hand two columns) over time of mean daily locations for: (A) an adult male with a settled home range; (B) an adult female with a shifting home range; (C) a sub-adult male showing exploratory movements, and; (D) a sub-adult male dispersing a long distance. Note the different scales for the y-axes.

Inspection of graphs of squared displacement over time identified distinct movement patterns among the 79 possums. There were four predominant movement types (Table 1): settled home range, home range displacement, exploratory movements or dispersal. These patterns are illustrated with data from four typical possums (Fig 2). In Fig 2A, the predominant movement pattern within a settled home range is shown for an adult male that had small, more-or-less random displacement distances, with little change in its elevation (520-600m) or distance to a river (270-400m). The next most common movement pattern (Fig 2B; home range displacement) is shown for an adult female shifting its home range, indicated by a steadily increasing displacement distance over time and no indication of return to a nominally consistent home range. At the same time, this animal moved away from the river and towards a higher elevation area. The third most common movement pattern (exploratory movement; Fig 2C) is shown for a sub-adult male which made forays over two 7-day periods. The long-distance moves largely followed a river course but varied little with elevation. The final movement pattern (Fig 2D) represented dispersal: this is shown for a sub-adult male, which moved ~ 2.5 km down a river valley over a period of approximately 3 days; having made this move the animal then remained in a localised area where, presumably, it had settled.

**Table 1:** Summary of age-sex classes and number (and percentage) of possums that showed each of the four types of movement patterns. Data are pooled from three sites in the Upper Ahaura Vector Control Area in 2010 and from two sites in the Whataroa–Waitangi Vector Control Area in 2011.

Age-sex class	Number	Settled home range	Home-range displacement	Exploratory movements	Long-distance dispersal
Adult male	23	22	1	0	0
Adult female	27	25	2	0	0
Subadult male	14	7	3	2	2
Subadult female	15	12	0	2	1
Total	79	66 (83.5%)	6 (7.6%)	4 (5.1%)	3 (3.8%)

Overall, most possums (83.5% of all possums, 94% of adults and 65.5% of sub-adults) exhibited a settled home range pattern (Table 1). Sub-adults recorded outside their initial home range were divided approximately evenly (10–14%) between the other three types of movement.

### Predicted movements across a buffer

**Fig 3:**
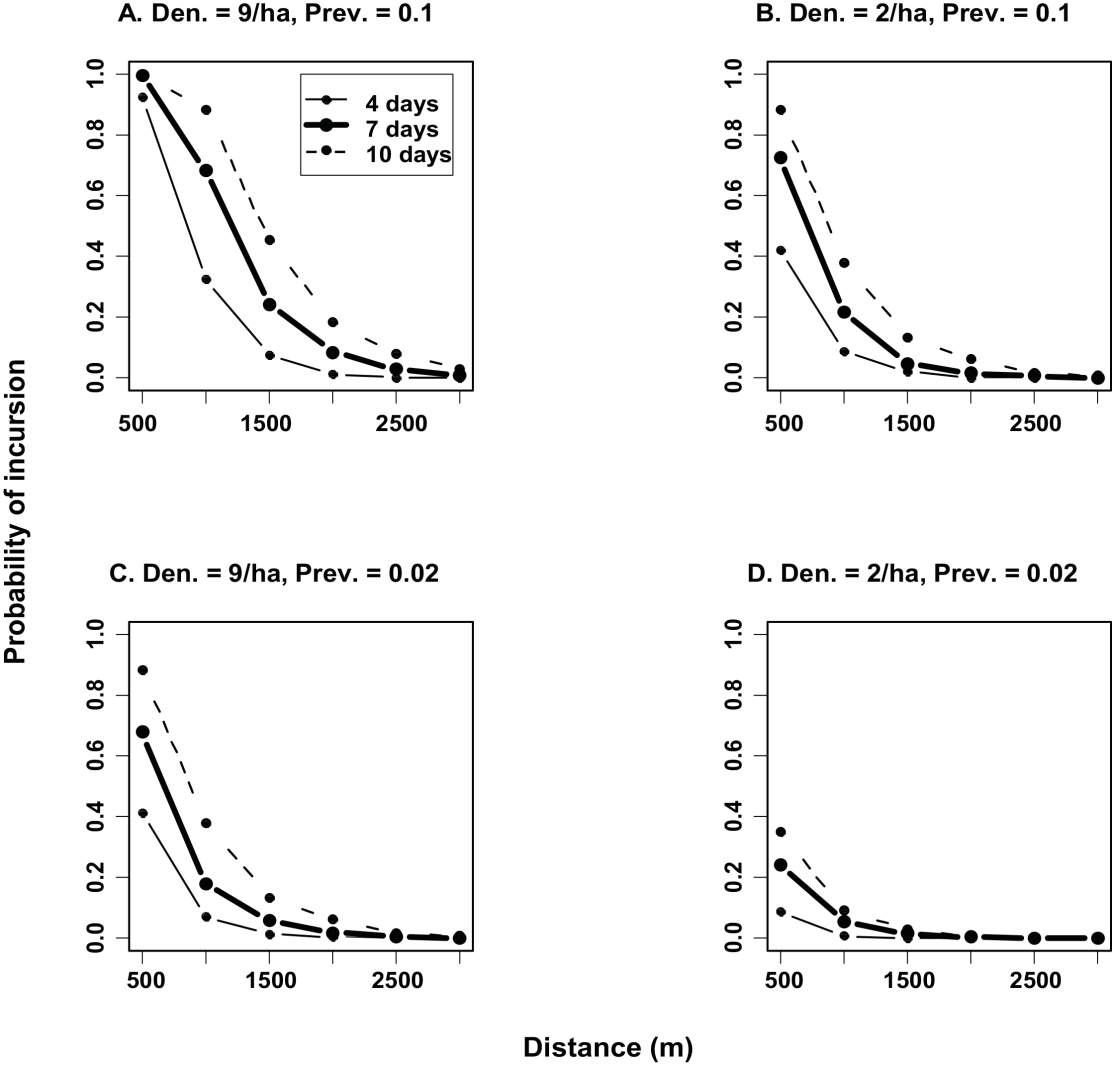
Predictions of the probability of a TB incursion event onto farmland (i.e. at least one *M*. *bovis*-infected possum crossing a buffer) during the period when most possum dispersal occurs. Simulations were run with buffer widths ranging from 500 – 3000m and with the following combinations of population density and disease prevalence in untreated forest: (A) density = 9 ha^-1^ and TB prevalence = 0.1; (B) density = 2 ha^-1^ and TB prevalence = 0.1; (C) density = 9 ha^-1^ and TB prevalence = 0.02; and (D) density = 2 ha^-1^ and TB prevalence = 0.02.

Based on the GPS data, the most likely duration of a representative movement event (i.e. a period that included dispersal or a bout of exploratory movements) was 7 days, while 4 and 10 days represented low and high estimates of the duration of such events. The probability of a TB incursion event (i.e. at least one infected possum traversing onto farmland) varied mostly with buffer width, and to a lesser extent with the population density and prevalence of TB in the source possum population (Fig 3). The probability of incursion increased with increasing number of days of the representative movement event. However, if priority was given to maintaining a very low probability of incursion (e.g. < 0.01), the outcomes were similar across the range of days in the simulation. In the best-case scenario, where density was 2 possums ha^-1^ and TB prevalence was 0.02 (Fig 3D), buffers of at least 2000m were not breached, irrespective of the number of days over which the simulated representative movement event took place. In the intermediate risk scenarios (Fig 3B and 3C), a 3000m buffer was predicted to be acceptable for all event durations. In the worst-case scenario (i.e. one likely to produce the most pessimistic estimate of the effectiveness of a buffer), in which density was 9 possums ha^-1^ and TB prevalence was 0.1 (Fig 3A), the 3000m buffer was predicted to be acceptable (< 0.008 probability of TB incursion) if the duration of the representative movement event was less than 10 days. If a 10 day event was simulated in this scenario, the probability of incursion with a 3000m buffer increased to 0.031.

## Discussion

Although the use of possum-free buffers is potentially a useful strategy for TB management in New Zealand, there have been few studies of the movements of possums, particularly sub-adults, across such buffers. We employed a two-stage process to assess the utility of buffers. First, we characterised the movements of adult and sub-adult possums into four typical patterns, three of which (home range displacement, exploratory movements or dispersal) would involve possums leaving their initial home range; and we identified the most likely duration of the last two types of movement events. Second, we used these data to simulate a possum population exhibiting these characteristic movement patterns to quantify the probability of a TB incursion onto farmland during a representative movement event lasting 4–10 days. These characteristic short-term movements were simulated to occur at any time during the time of year when our data were collected, and thus represent the probability of a TB incursion over the peak dispersal period for possums (3–6 months).

The utility of buffer zones for TB control in New Zealand has been discussed previously [13], and while it was known at the time of that earlier study that the optimal extent and frequency of possum control applied to buffers could be approximated using an existing simulation model [10], the lack of sufficiently detailed knowledge of possum movement patterns, particularly for sub-adults, required some unrealistic, but critical, simplifications of the parameters of that model [39,40]. In particular, modelled possum home ranges remained fixed even if nearby possums were removed in the simulations; adults and sub-adults were considered to disperse at equal rates (10% of males and 5% of females per year); and habitat attributes and topographic features were not considered to influence modelled dispersal distances or directions. Some of these assumptions have been addressed in the present study: for example, we identified that 16.5% of possums exhibited some movement beyond their initial home range which could result in animals moving into, and possibly across, a recently depopulated buffer. This particular finding is supported by other recent studies using samples of possums fitted with GPS telemetry units [41,42].

In the previous study cited above, Pech et al. [13] compared rates of reinvasion by possums for a simulated ‘vaccine-protected’ buffer (i.e. a buffer zone with an intact possum population) with reinvasion rates for a buffer in which possums had been controlled by conventional means (i.e. a buffer zone in which the possum population had been reduced by poisoning). In that study, there was relatively little movement of adult possums from native forest at carrying capacity (>3 possums/ha) into a forest buffer that had received recent possum control: over the first 6 months of monitoring, a poison-treated buffer c. 350 m wide was predicted sufficient to contain 95% of movements by adult possums, while a buffer c. 450 m wide would have been equally effective for an additional 6-month period. In the present study we also included sub-adult possums in our simulations, i.e. animals that (along with adults) were thought at the outset to represent the greatest risk of translocating TB during dispersal [27]; even then, modelling predicted that, for a region with low possum density and low TB prevalence, there would be a very low probability of TB incursion onto farmland during a representative possum movement event lasting 4–10 days. Since buffer widths used in practice for TB containment in forested areas currently range from 5 – 15km [8], the present results suggest there is scope for buffer widths to be reduced while still maintaining good protection against TB incursions due to short-term movements that typify possum dispersal or exploratory behaviour.

Results from the present study have addressed additional issues related to the movement patterns of possums. Despite the fact that the forest buffers at our study sites provided vacant habitat just as suitable for possums as untreated forest, the directions of dispersal and exploratory movements by possums did not appear to be biased towards these buffers. Further, our results suggest that, overall, dispersal was uncommon for sub-adults (in contrast to the results of Cowan & Clout [5] who identified dispersal among 25% of juvenile males and 10% of juvenile females); and that dispersal was rare among adult possums, despite this group being regarded as potential dispersers [6]. The reasons for the lack of dispersal in our study remain unknown; however, the minimal shift in home range by most animals (including sub-adults) suggests that as residents they were not resource-limited at our study sites. It is unlikely that home range boundaries were influenced strongly by social interactions, because we found no evidence to support preferential movement of possums into vacant territory (however, home range expansion due to localised decreases in density has been reported for possums in different habitat, such as low-canopy native lowland forest in New Zealand [43] and old-growth eucalyptus forest in Australia [44]). Finally, based on possums’ GPS locations in relation to topography, we found no evidence that forested ridgelines changed movement patterns of possums, although our data did suggest that large rivers could act as barriers. Size of river flow has been identified recently as a major factor impeding possum dispersal [15], possibly sufficient to affect subsequent population mixing [45].

Our study has improved knowledge on the movements of possums, particularly for sub-adults, and how this may affect potential movement of an *M*. *bovis*-infected possum across a buffer zone; but it did not capture some aspects of the behaviour of possums that may influence intra-specific disease transmission [46]. In particular, the possum:possum contact rate probably varies depending on habitat and population density, and also social structure of the population [47] but this is a variable that cannot be modelled easily at present. Experience from large-scale population control of *M*. *meles* in Great Britain has shown that perturbation of social structures among badger populations, by localised lethal control, can affect the animals’ movements [48], with potentially adverse consequences for the spread of wildlife-transmitted TB [49].

Possum-depopulated buffer zones continue to be used in New Zealand [8] to isolate a source of heightened infection risk (e.g. forested habitat containing possums with a recent history of TB) from a nearby asset (TB-susceptible livestock on farmland). Similarly, buffer zones have been used successfully worldwide for the control of other diseases, such as epidemics of foot and mouth disease [50], and for the containment of sylvatic rabies outbreaks in new wildlife disease foci [51] and in urban areas with high human density [52]. Our study suggests that the types of movement by possums (dispersal and exploratory movements) most likely to transfer *M*. *bovis* infection across a buffer are uncommon and that current buffer widths in New Zealand represent a risk-averse management strategy. However, gaps remain in our knowledge of the epidemiology of TB (especially factors affecting disease transmission rates) in possum populations re-colonising a forest buffer. We further conclude that in establishing buffer zones for protection of livestock against wildlife TB there is potential to take advantage of natural features, such as large rivers, that can block possum movements and divert them to areas where more intensive control can be conducted.

## Supporting Information

S1 File Methodological details of possum handling and collar fitting (including animal numbers), information relating to the interpretation of trap-catch data for possum population monitoring, and relevant TB information. (DOCX)
