# Supplementary material for: Assessing Movements of Brushtail Possums (Trichosurus vulpecula) in Relation to Depopulated Buffer Zones for the Management of Wildlife Tuberculosis in New Zealand
Source: PLoS One. 2015 Dec 21;10(12):e0145636. doi: 10.1371/journal.pone.0145636 (PMC4686990; doi:10.1371/journal.pone.0145636)
Supplement: S1 File — (DOCX) [file pone.0145636.s001.docx]

**Methodological details of possum handling and collar fitting (including animal numbers), information relating to the interpretation of trap-catch data for possum population monitoring, and relevant TB information.**

*Possum handling and collar fitting*

For live manipulation, possums were first sedated by intra-muscular injection of ketamine hydrochloride (0.1ml/kg). One of three types of GPS/VHF collar was then fitted to the possums: large heavier collars (c.130 g) for adult-sized animals (~2.5kg), medium-sized collars (c. 106 g) for adults and larger subadults (~>1.5kg), and small ‘microGPS’ collars (c. 64 g) for smaller subadults (<1.5kg). A cotton weak link and foam were added to the collars used for subadults so that any animals not recaptured would have room to grow, and collars were deployed during a period when there was likely to be little or no change in neck dimensions [1]. All collared possums were also fitted with numbered eartags so that an animal could be identified if it was re-caught without its collar. The collars were automated to receive and store on-board GPS location data, and were set-up to acquire their first datum early in the night (between 9pm and 11pm, depending on the timing of full nightfall) and at subsequent 4-hourly intervals throughout the night. Additional GPS fix points were also recorded manually whenever a possum was tracked by foot or by air to a daytime den site (approximately monthly, to assess continued functional status of equipment and to verify survival), and also whenever a possum was trapped or retrieved. Upon final capture (or poisoning) the age-sex class of each possum was established more precisely by measuring its weight and recording its tooth wear [2-5] and by evaluating the stage of development of the pouch or testes. All animal handling procedures were conducted under guidance from the Landcare Research Animal Ethics Committee (Lincoln, New Zealand; project approval number 09/09/02).

*Data acquisition*

In the Upper Ahaura Vector Control Area, 52 of 58 collars deployed were retrieved at the end of the study (38 adult-sized collars and 14 microGPS collars). GPS data were able to be downloaded from 16 adult female and 8 subadult female possums, and from 16 adult male and 9 subadult male possums. Of these, 46 possums had at least 8 days of location data that could be used in the analyses. All the possums at the Haupiri site were caught initially and recaptured in mixed beech–kāmahi–podocarp forest. At the Waikiti Hut site, three possums were caught initially beside grassy areas and the remainder in forest, but all were recaptured in beech–kāmahi forest. At the Waiheke site, four possums were caught initially beside grassy river flats and two were recaptured in this habitat; the rest were caught and recaptured in beech–kāmahi forest. Trap-catch rates for the three sites were estimated from the initial live-capture data (corrected for sprung traps); however, these estimates were based on a trapping effort different to the protocol for the standard Trap-Catch Index (see below), i.e. more traps per line with fewer set lines than the standard protocol requires, and using soft catch traps set on ‘best sign’ [6]. Trap-catch rates were similar for all sites: 11.26% (number of possums captured per 100 trap nights) at Haupiri, 11.85% at Waikiti Hut and 13.32% at Waiheke.

In the Whataroa–Waitangi Vector Control Area, 38 of the 45 collars deployed were recovered at the end of the study (18 from the Whataroa site and 20 from the Wanganui site). Of these, 33 were used in the analysis as they had at least 8 days of location data. These consisted of 8 collars from adult males, 12 from adult females, 5 from subadult males and 8 from subadult females. At the Wanganui site all collared possums were trapped in mixed podocarp–kāmahi forest and all were subsequently retrieved in the same forest type. At the Whataroa site, most possums were caught initially in mixed podocarp–kāmahi forest, three near grassy flats, and seven at the river edge, in a gully or on an island in the river. All the Whataroa possums that were retrieved were caught in the mixed podocarp–kāmahi forest. Trap-catch rates (possums per 100 trap nights) were 17.8% for the Whataroa site and 4.4% for the Wanganui site.

*Interpretation of trap-catch indices*

Generally the density of possum populations is quantified and expressed in relative terms, often as an index. True abundance measures (absolute densities) are more difficult, costly and time-consuming to obtain, and for possum-management purposes pest-control agencies usually express relative indices of abundance. One such measure is the Trap-Catch Index (TCI) which expresses the proportion of standard leg-hold traps set that capture possums over a set period of time. To achieve this in a uniform fashion, traps must be set at specified frequency and spacing, and in a prescribed manner, according to nationally-recommended practices (the methods for obtaining and calculating possum TCI are described in detail in an online publication [6]).

Although of variable precision, the relative abundance measure of TCI can be approximated to a true abundance measure, according to methods described by Ramsey et al. [7]. While this is seldom used in practice, it is a useful ‘real world’ measure, particularly for non-practitioners to obtain an approximation of true possum densities. For example, a TCI of 10% (i.e. 10 possums captured per 100 trap nights) approximates to 2 possums per ha [8], but at low-end values (i.e. TCI < 2%) the estimate becomes imprecise and conversion values are often given as a below-limit description, e.g. while a TCI of 1% approximates to 0.1 possums/ha it would probably be stated as representing “a low density possum population comprising < 0.5 possum/ha”.

Changes to the way the relative index of possum abundance is measured in the field will affect the precision of the estimate. In the context of the present study, the trap-catch protocol used to assess relative possum abundance, at two sites in the Upper Ahaura VCA following poison control, used standard traps set in a non-standard position raised above the ground (in this case to prevent accidental bycatch of ground-dwelling native birds). Such raised trap sets have been reported previously to exhibit equivalent or lower trapping success compared to standard leg-hold trap sets placed on the ground [9]. The implication for this study is that while the two post-poison TCI values recorded and reported of <1.5% and <0.5% would ordinarily suggest extremely low possum densities of <0.2 possums/ha, these values may be underestimations, and both the use of a low-efficiency trap-set [9] and the imprecision of low-end relative:absolute abundance conversions [7] would urge caution in this statement. Hence the conclusion in the manuscript was that possum density, post-control, was below 0.5 possums/ha (but how much lower cannot be stated with confidence). Similarly, relative possum abundance indices prior to poison control had been assessed at three sites in the Upper Ahaura VCA as 11.3%, 11.8% and 13.3% (which would ordinarily indicate a population of 3 – 4 possums/ha); however that trapping protocol used more traps per line with fewer set lines than the standard protocol stipulates. Hence the true possum abundance prior to control was not known precisely.

*TB status among possum populations in the study areas*

Typical values for TB prevalence among possums in New Zealand vary with habitat. In forested habitat where TB is long-established in the local possum population, prevalence is usually <5% and often only 1–2% [10]. However TB prevalence can be substantially higher than this (in the range 9–32%) within local clusters of infection (so called ‘hot-spots’) and has been reported to exceed 60% in some foci [11].

Surveys for TB prevalence among possum populations are conducted in VCA regions on an ‘as required’ basis under the national pest management plan. Such data are collated for pest control management purposes but are seldom published in a peer-review format (and hence publicly accessible). Nevertheless, for regions studied here, TB data had been reported for possums in the Upper Ahaura VCA from prior studies in the mid- to late-1990s [12, 13]. Over four consecutive years TB prevalence fluctuated from 11%, to 10%, then to 4% and finally to 2% [12]. The highest extreme point prevalence of 53% was recorded in 1992 [13]. Temporal fluctuations of disease levels within possum populations, such as these, are common [10], and in general disease will persist in an endemic region until the possum population is controlled.

The TB status of possums fitted with GPS collars in the present studies was determined at two points: at the initial fitting of the collar and upon subsequent recovery of the collar from the carcass at completion of the study. On both occasions, external palpation of possums did not detect swellings in the axillary, inguinal or abdominal regions that would indicate lymphadenomegaly representative of progressive TB [14]; and visual inspection of the body did not detect cases of ruptured fistula or draining sinuses that would be indicative of advanced, fulminating disease [15]. However, TB evaluation in the present studies did not involve the more detailed practice of carcass necropsy, collection of predilection site tissues and mycobacteriological culture that is now standard for TB assessment in possums [16,17]. Hence it is possible that some possums still alive immediately prior to study completion may have harboured sub-clinical *M. bovis* infection (NB: any with sub-clinical infection at study commencement would have progressed to overt and readily-detectable disease by study completion; 14,15]).

*References cited in Supplementary Information*

[1] Cowan PE, White AJ. Evaluation of a tooth-wear age index for brushtail possums, *Trichosurus vulpecula*. Austr Wildl Res 1989; 16: 321–322.

[2] Glen AS. Animal pests: residual trap catch index for possums. Inventory and monitoring toolbox publication for animal pests no. DOCDM-1414383. Department of Conservation, Wellington, New Zealand; 2014. Pp 1 – 14.

[3] Kean RI. Growth of the opossum (*Trichosurus vulpecula*) in the Orongorongo Valley, Wellington, New Zealand 1956–1961. N Z J Zool 1975; 2: 435–444.

[4] Kingsmill E. An investigation of criteria for estimating age in the marsupials *Trichosurus vulpecula* Kerr and *Perameles nasuta* Geoffrey. Austr J Zool 1962; 10: 597–617.

[5] Lyne AG, Verhagen AMW. Growth of the marsupial *Trichosurus vulpecula* and a comparison with some higher mammals. Growth 1957; 21: 167–195.

[6] NPCA. Possum population monitoring using the trap-catch method. National Possum Control Agencies publication, Wellington, New Zealand; 2008. Pp 1 -14.

[7] Ramsey DSL, Efford MG, Ball S, Nugent G. The evaluation of indices of animal abundance using spatial simulation of animal trapping. Wildl Res 2005; 32: 229–237

[8] Thomas MD, Brown JA. Possum monitoring using raised leg-hold traps. Science for Conservation series publication no. 164. Department of Conservation, Wellington New Zealand; 2000. Pp 1 – 17.

[9] Winter JW. Tooth wear as an age index in a population of the brushtailed possum, *Trichosurus vulpecula* (Kerr). Austr Wildl Res 1980; 7: 359–369.

[10] Coleman J, Caley P. Possums as a reservoir or bovine Tb. In: Montague TL ed. The brushtail possum: biology impact and management of an introduced marsupial. Manaaki Whenua Press, 2000. Lincoln, New Zealand. Pp. 92–104.

[11] Nugent G, Buddle B, Knowles G. Epidemiology and control of Mycobacterium bovis infection in brushtail possums (*Trichosurus vulpecula*), the primary wildlife host of bovine tuberculosis in New Zealand. N Z Vet J 2015; 63 (S1): 28-41.

[12] Coleman JD, Cooke MM, Jackson R, Webster R. Temporal patterns in bovine tuberculosis in a brushtail possum population contiguous with infected cattle in the Ahaura Valley, Westland. N Z Vet J 1999; 47: 119-124.

[13] Coleman JD, Jackson R, Cooke MM, Grueber L. Prevalence and spatial distribution of bovine tuberculosis in brushtail possums on a forest-scrub margin. N Z Vet J 1994; 42: 128-132.

[14] Nugent G, Whitford EJ, Yockney I, Perry M, Tompkins DM, Holtslag N, Cross ML. Percutaneous interdigital injection of *Mycobacterium bovis* as a model for tuberculous lesion development in wild brushtail possums (*Trichosurus vulpecula*). J Comp Pathol 2013; 148: 33-42.

[15] Nugent G, Yockney I, Whitford J, Cross ML. Mortality rate and gross pathology due to tuberculosis in wild brushtail possums (*Trichosurus vulpecula*) following low dose subcutaneous injection of *Mycobacterium bovis*. Prev Vet Med 2013; 109: 168-175.

[16] Warburton B, Livingstone P. Managing and eradicating wildlife tuberculosis in New Zealand. N Z Vet J 2015; 63 (S1): 77-88.

[17] Anderson DP, Ramsey D, de Lisle GW, Bosson M, Cross ML, Nugent G. Development of integrated surveillance systems for the management of tuberculosis in New Zealand wildlife. N Z Vet J 2015; 63 (S1): 89-97.
